# Recent Advances in Metal–Organic Framework-Based Nanozymes for Enhanced Biosensing Applications

**DOI:** 10.3390/bios16040197

**Published:** 2026-04-01

**Authors:** Jianping Wang, Xiaoying Zhou, Zhonghao Dai, Lu Xu, Siyu Nie, Dongjie Yang, Yi Yang, Liyuan Wang, Jiayun Yao, Zihong Ye

**Affiliations:** 1 Zhejiang Provincial Key Laboratory of Biometrology and Inspection & Quarantine, College of Life Sciences, China Jiliang University, Hangzhou 310018, China; p22091055082@cjlu.edu.cn (X.Z.); s23090710005@cjlu.edu.cn (Z.D.); p24091055069@cjlu.edu.cn (L.X.); p24091055044@cjlu.edu.cn (S.N.); s24090710062@cjlu.edu.cn (D.Y.); p23091055067@cjlu.edu.cn (Y.Y.); p24091055057@cjlu.edu.cn (L.W.); 2 Key Laboratory of Microbiological Metrology, Measurement & Bio-Product Quality Security, State Administration for Market Regulation, China Jiliang University, Hangzhou 310018, China; 3 Agriculture Ministry Key Laboratory of Healthy Freshwater Aquaculture, Key Laboratory of Fish Health and Nutrition of Zhejiang Province, Zhejiang Institute of Freshwater Fisheries, Huzhou 313001, China; yaojiayun@126.com

**Keywords:** metal–organic frameworks, nanozymes, nanocomposites, catalysis, biosensors

## Abstract

In recent years, with the rapid development of materials science, there has been a significant increase in the focus on nanozymes. Metal–organic framework (MOF)-based nanozymes are a class of porous organic–inorganic coordination materials capable of mimicking the catalytic activity center of natural enzymes. The properties of MOF-based nanozymes include high specific surface area and porosity, structural diversity and customizability, and excellent catalytic activity and stability. Through rational design, the activity of MOF-based nanozymes can be further enhanced to promote their application in biosensing and other fields. This paper systematically investigates the intrinsic relationship between the structure of MOFs and their catalytic performance, with a focus on the diverse catalytic activities of MOF-based nanozymes, including peroxidase, oxidase, catalase, superoxide dismutase, and hydrolase. It reviews optimization strategies for the key parameters, such as selectivity and stability, summarizing the advances in synthesis strategies. Furthermore, the application progress of MOF-based nanozymes in the field of biosensing is reviewed, covering areas such as biomarker detection, virus recognition, and the screening of pathogenic microorganisms, among others. This review provides a systematic discussion of the opportunities and challenges in the development of MOF-based nanozymes, identifies the key scientific issues that are driving the field forward, and offers important references for optimizing MOFs’ structural designs and developing high-efficiency biochemical sensors.

## 1. Introduction

Natural enzymes, renowned for their exceptional catalytic activity and substrate specificity, serve as indispensable biocatalysts in living systems by accelerating biochemical reactions with unparalleled efficiency [1,2]. However, their widespread industrial application faces formidable challenges, including prohibitive purification costs, intrinsic instability under extreme pH/temperature conditions, and susceptibility to proteolytic degradation—limitations that frequently lead to irreversible activity loss [3]. These constraints have spurred intensive research into artificial enzyme mimics that can replicate natural enzymatic functions while overcoming these inherent drawbacks [4,5]. Nanozymes represent a class of engineered nanomaterials that exhibit intrinsic enzyme-mimicking catalytic functionalities. Recent advances in nanotechnology have facilitated the development of nanozymes—a class of nanomaterial-based enzyme mimics that integrate sophisticated catalytic mechanisms with robust structural properties [6,7,8].

**Figure 1:**
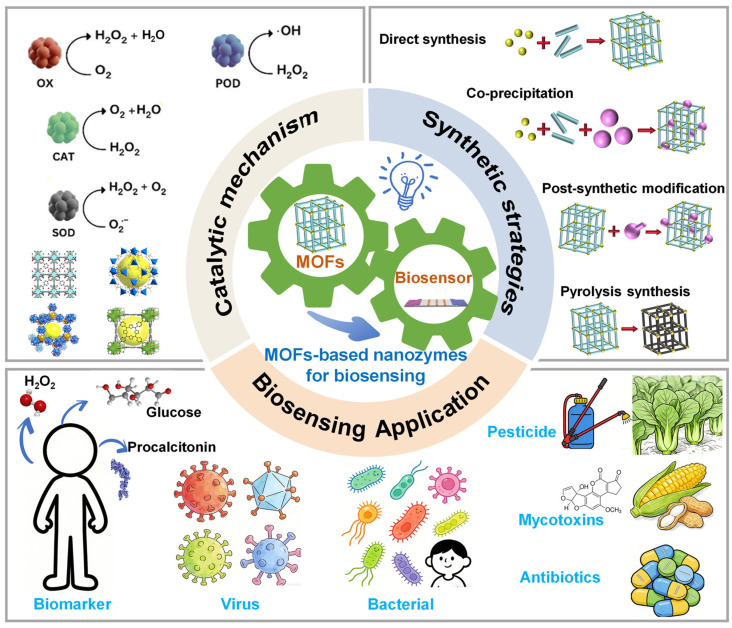
Schematic representation of MOF-based nanozymes in biosensing: catalytic mechanism, synthetic strategies and applications.

Since the discovery in 2007 that Fe_3_O_4_ nanoparticles can act as peroxidase analogues [9], the concept of nanozymes has rapidly attracted significant attention across interdisciplinary fields, and their unique catalytic properties have sparked extensive research in areas such as biosensing, biomedicine and food safety detection [10,11,12]. With the rapid progress and development of nanotechnology, emerging nanomaterials have shown significant potential as a basis for building a versatile and sensitive biosensing platform. Among these nanomaterials, MOFs represent a class of inorganic–organic hybrid materials formed by the coordination of metal ions/clusters and organic bridging ligands [13,14,15]. MOFs are a class of two-dimensional or three-dimensional ordered porous crystalline materials formed by the self-assembly of secondary structural units with multi-dentate organic linkers. Their main components include metal cations and their salts as nodes and organic bridging ligands with coordination functions. The three-dimensional channels within the material and the highly dispersed active sites together form a microreactor-like structure [16,17,18,19]. The spatial confinement not only facilitates rapid substrate transport but also optimizes product diffusion pathways through the ordered channel network, thereby enhancing catalytic cycle efficiency. MOF-based nanozymes exhibit unique structural features—such as atomic/molecular-level catalytic active sites, ultra-high porosity, exceptionally large specific surface area, and excellent loading capacity within their crystal frameworks—which significantly enhance their catalytic activity and selectivity, thereby enabling their extensive application in sensing fields (Figure 1). As extensively reported in the recent literature, the stability enhancement provided by MOF-based enzymes is comprehensive and multidimensional, encompassing chemical stability, thermal stability, and operational stability [20,21], specifically: (1) The rigid porous framework of MOFs can shield enzymes from damage in harsh chemical environments, such as extreme pH and organic solvents. (2) The inherent excellent thermal stability of MOFs, especially zirconium-based MOFs, contributes to improving the thermal tolerance of composites. (3) MOFs significantly improve operational stability, namely long-term storage stability and reusability.

In comparison with other nanozymes, such as carbon-based and metal nanozymes, MOF-based nanozymes exhibit significant advantages, including exceptional structural designability (Table 1). The catalytic activity of MOF-based nanozymes is primarily attributable to specific active sites within their structures. The most common and direct sources of catalytic activity include metal nodes/clusters, organic ligands possessing redox activity or specific functional groups, active species introduced via doping or post-synthesis modification, and coordination-unsaturated metal sites arising from defects [22]. However, MOF materials typically exhibit extremely low electrical conductivity (approximately 10^−8^ to 10^−10^ S cm^−1^) or even insulating properties. This is primarily attributed to their lack of low-energy charge transfer pathways and effective free carrier transport channels. The limited conductivity of these materials severely restricts their potential applications in the domain of electrochemistry [23]. Studies have identified that conductivity influences the charge transfer patterns, which are primarily categorized into two mechanisms: (1) a redox hopping mechanism between organic linkers and (2) a guest-promoted pathway involving host–guest interactions between the inorganic framework nodes and guest molecules [24].

Enhanced conductivity facilitates easier access of electrons to active sites, thereby improving the utilization of active sites and boosting performance. An increasing number of highly conductive MOFs have been reported to date, which can be broadly classified into three categories: intrinsically conductive MOFs, MOF composites and MOF derivatives [25]. Among these, the research on MOF composites has been relatively extensive, demonstrating their favorable electrical conductivity, such as by constructing composite structures with carbon materials or introducing conductive polymers. For example, Hyun Kim et al. introduced surfactants to mitigate Z-axis stacking in zinc–porphyrin MOFs, resulting in the formation of ultrathin two-dimensional nanosheets, into which carboxyl-functionalized multi-walled carbon nanotubes were embedded. This structure preserved the layered characteristics of MOFs while promoting the formation of abundant hierarchical micropores and conductive pathways, thereby enhancing conductivity [26].

A further critical challenge associated with MOF-based nanozymes lies in their pronounced pH-dependent catalytic activity. Their high efficiency is typically confined to a narrow pH window, which often starkly mismatches the physiological microenvironment of biological samples. To address this limitation, researchers have recently developed regulatory strategies—such as reconstructing the catalytic microenvironment or reshaping the reaction mechanism—aimed at enabling nanozymes to overcome pH constraints [27]. For example, Wei et al. proposed a microenvironment engineering strategy [28]. In that study, MOFs were employed as carriers, thereby confining low-molecular-weight polyacrylic acid (PAA) as a Brønsted acid within their pores. The carboxyl groups of PAA have been shown to sustainably release protons, thereby establishing a localized, stable acidic microenvironment within MOFs. This design facilitated the maintenance of the MOF-based nanozyme’s catalytic sites within the optimal acidic conditions required for peroxidase-like activity, even when situated in a neutral bulk environment (e.g., pH 7.4). Consequently, it exhibited high catalytic activity, effectively resolving the efficiency bottlenecks caused by pH mismatches in cascade reactions and significantly enhancing the overall catalytic efficiency. Wang et al. developed a dual-ligand Cu-MOF nanozyme in which histidine-induced distortion of Cu(II) coordination innovatively alters the H_2_O_2_ decomposition pathway, thereby overcoming the conventional pH limitation [29]. This nanozyme exhibits catalytic activity across a broad pH range of 2.0–12.0, with optimal performance at pH 9.0. Under alkaline conditions, its catalytic efficiency surpasses that of previously reported nanozymes by 2–3 orders of magnitude.

In summary, MOF-based nanozymes have become a significant platform for mimicking natural enzymatic functions, owing to their precisely tunable structures and abundant catalytic sites. Despite challenges such as pH sensitivity and limited conductivity, strategies including microenvironment engineering and the construction of conductive composite structures have substantially enhanced their catalytic performance, thereby promoting their practical applications in biosensing, medical diagnostics, and related fields.

**Table 1:** Comparative analysis of MOF-based nanozymes versus conventional nanozymes.

	MOF-Based Nanozymes [14,15,16,17,18,30]	Carbon-Based Nanozymes [31,32,33]	Metal Nanozymes [34,35,36]
**Material composition**	Metal ions/clusters and organic ligands form porous crystalline structures through coordination bonds.	Carbon materials, such as fullerenes, graphene and carbon quantum dots.	Precious metals (Au, Pt), transition metals (Fe, Co), and metal oxides (Fe_3_O_4_, CeO_2_).
**Structural designability**	The structure of MOFs exhibits exceptional designability. By varying the types of metal nodes (single or bimetallic) and organic linkers, precise control over their pore size, surface chemistry, and active sites can be achieved.	The structure can be varied, with the electronic structure being tunable via the addition of heteroatoms (e.g., N, S).	These materials generally exist as nanoparticles with simple architectures, enabling property modulation through precise control of their dimensions and shapes.
**Catalytic performance**	The most abundant sources of activity include intrinsic activity (metallic nodes/unsaturated sites), encapsulated/composite activity (loaded metal nanoparticles or native enzymes), and derived material activity (highly reactive carbonaceous or metallic compounds formed via pyrolysis).	The catalytic activity of these surfaces is understood to originate from surface defects, edge effects, and dopant sites. The type and density of surface functional groups have been demonstrated to be pivotal in determining catalytic activity.	While certain single-atom metal nano-enzymes have been demonstrated to exhibit catalytic activity that approaches that of natural enzymes, their substrate selectivity remains comparatively poor.
**Major limitations**	Catalytic activity is significantly affected by pH, and electrical conductivity is poor.	The regulation of catalytic activity is challenging, and the density of active sites is relatively low.	The inherent tendency towards aggregation, metal ion leakage, relatively high cost (due to the use of precious metals), poor selectivity and limited functionality are significant drawbacks.

## 2. Classification of Representative MOF-Based Nanozymes

Since it was proposed in 2013 that nanomaterials can simulate five key redox enzymes (including peroxidase, hydrolase, oxidase, catalase and superoxide dismutase), the development of new nanozymes has become a key research direction in this field [37,38,39]. Since the discovery of the catalytic properties of nano-enzymes, this research field has expanded from the initial redox reactions system to multiple types of catalytic systems, such as hydrolysis reactions. To date, hundreds of nanomaterials have been proven to have catalytic activities similar to those of natural enzymes. This article will conduct a systematic review of representative MOF-based nanozymes in various enzymatic reactions, with a focus on analyzing their catalytic mechanisms and structure–activity relationships.

### 2.1. Peroxidases

In recent years, a large number of research reports have shown that iron-containing MOFs have peroxidase catalytic activity, and their catalytic principle is usually a Fenton-like mechanism; that is, hydrogen peroxide reacts with ferrous ions to produce hydroxyl radicals with strong oxidizing capacity [40,41]. The peroxidase-like activity of such typical iron-based MOFs relies on the Fe^3+^/Fe^2+^ redox cycle. The acidic environment provides ample protons, which stabilize the high-valent iron–oxo intermediates and facilitates electron transfer, thereby efficiently driving the catalytic cycle. The core mechanism of peroxidase catalysis resides in the activation of hydrogen peroxide. Most peroxidase nanozymes exhibit optimal activity under acidic conditions; in an acidic environment, hydrogen peroxide is more prone to protonation [28]. This process facilitates the weakening of its O-O bond, making it more susceptible to cleavage by the metal active centers of nanozymes, thereby generating reactive oxygen species, such as highly reactive hydroxyl radicals. It is worth noting that the catalytic activity is not limited to iron-based materials, and transition metal nanozymes, such as copper- and zirconium-based nanozymes, can also exhibit peroxidase-like functions through a similar free radical chain reaction pathway [42,43,44,45]. Peroxidase catalyzes the initial step of the reaction, whereby the amino group of 3,3′,5,5′-tetramethylbenzidine (TMB) loses an electron to a cationic radical in conjunction with hydrogen peroxide. This results in the formation of a blue dimer charge transfer complex within the system. Subsequently, at low pH, the complex will lose one electron and form a stable quinone-conjugated monomer structure, which will eventually turn yellow.

**Figure 2:**
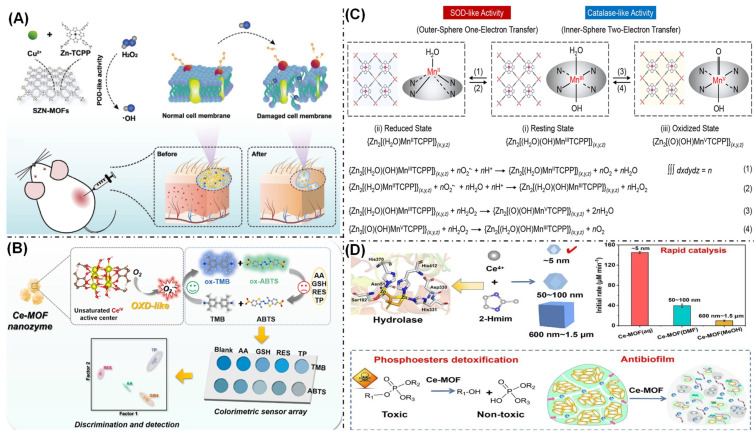
(**A**) Schematic of peroxidase-like activity of a single-zinc-site MOF nanozyme [46]. (**B**) Mechanistic oxidase-like activity of a hexanuclear cerium-based MOF nanozyme [47]. (**C**) Schematic of the interconversion pathways among the resting, reduced, and oxidized states of a ZMTP nanosheet, along with the detailed chemical reactions for each step [48]. (**D**) Schematic of the synthesis and application of ultra-small Ce-MOFs with hydrolysis enzymes-like activity [49].

In comparison with natural peroxidases, this class of MOF-based nanozymes exhibit enhanced peroxidase-like activity, superior stabilization, and elevated stability. As shown in Figure 2A, Wang’s group reported the surfactant-assisted synthesis of a single-zinc-site nanozyme (SZN-MOFs) using two-dimensional MOFs as carriers, achieving a zinc atomic loading of up to 4.6 wt.% [46]. The theoretical calculations and experimental results demonstrated that the as-prepared SZN-MOFs exhibited remarkable peroxidase-like activity, efficiently catalyzing the conversion of hydrogen peroxide into hydroxyl radicals, which was attributed to the high-density zinc single-atom active sites and the ultrathin structure of the two-dimensional MOFs. In a wound biofilm infection model, the SZN-MOFs significantly promoted wound healing and demonstrated high biocompatibility.

### 2.2. Oxidases

Oxidases catalyze the oxidation of substrates (electron donors) to the corresponding oxidation products in the presence of oxygen (electron acceptors) [50]. This process occurs simultaneously with the generation of H_2_O or H_2_O_2_. The advantage of such MOF-based nanozymes lies in their ability to directly catalyzes the oxidation of substrates (e.g., TMB) using the reactive oxygen species generated during the reaction, without the need for H_2_O_2_ involvement. This renders them safe and convenient to operate, thereby enabling their widespread application in the field of biosensing. In addition, many MOF-based nanozymes, such as Ce-based, Co-based, and Mn-based nanozymes, also exhibit oxidase catalytic activity [51,52,53]. For example, Li’s group developed a hexanuclear cerium-based MOF that exhibited remarkable oxidase-like activity (0.97 U mg^−1^) under neutral pH conditions, demonstrated ultra-high affinity toward the TMB substrate (K_m_: 0.012 mM), and maintained stable activity within 0–50 °C (Figure 2B) [47]. Both the theoretical calculations and experimental verification confirmed that its high catalytic efficiency originated from the unsaturated Ce^4+^ active sites and the unique Ce^4+^/Ce^3+^ redox cycling. These two factors synergistically promote the generation of superoxide radicals (O_2_•^−^) and significantly reduce the energy barrier of the rate-determining step, thereby enhancing the reaction kinetics. The catalytic activity of MOF-based nanozymes is primarily governed by their exposed active sites, composed of structurally simple metal ions or clusters. While MOF pore environments are tunable, they lack the precision of natural enzymes, which feature sophisticated three-dimensional architectures and refined active pockets for enhanced substrate specificity. The differential mass transfer efficiencies of substrates within the pores can also induce catalytic preference. Moreover, in complex matrices such as biological fluids, proteins and biomacromolecules often cause pore blockage or nonspecific active-site binding, compromising catalytic efficiency and selectivity for the intended substrate [19].

### 2.3. Catalases

A catalase is an antioxidant enzyme that is widely present in living organisms [54]. Its primary function is to catalyze the decomposition of H_2_O_2_ into water and oxygen, thereby removing H_2_O_2_ from the body and protecting cells from its toxic effects. MOF-based nanozymes, such as Mn based and Ce based, demonstrate higher catalytic activity and stability than natural catalases [42,55]. The catalytic reaction mimicked by MOFs for catalase activity centers on the disproportionation of H_2_O_2_ into H_2_O and O_2_. This process typically involves the redox cycling of metal centers, with each step accompanied by proton transfer [37]. This mechanism clearly demonstrates that protons (H^+^) are essential participants in the reaction. Only within a specific pH range is the concentration of H^+^ in solution sufficient to effectively drive the proton-coupled electron transfer steps, allowing the catalytic cycle to proceed smoothly. Su’s team synthesized two-dimensional iron-doped carbon nanosheets (Fe-N800 CS) with catalase-like activity by doping iron into zinc-based metal–organic frameworks (Zn-MOFs) and incorporating graphitic carbon nitride (g-C_3_N_4_) [56]. The as-prepared material demonstrated highly efficient catalase-mimetic activity, excellent reusability, and remarkable storage stability. Based on this nanomaterial, the team constructed a ratiometric fluorescent biosensing system that achieved highly sensitive detection of alkaline phosphatase and ascorbate oxidase, confirming its significant application potential in the biosensing field.

### 2.4. Superoxide Dismutase

Superoxide dismutase (SOD) is an antioxidant metalloenzyme found in diverse organisms that catalyzes the conversion of superoxide anions into H_2_O_2_ and oxygen O_2_ [57,58,59]. Previous studies have shown that it plays a key role in maintaining the balance between oxidation and antioxidation in the body. The catalytic activity of natural SOD stems from the metal ions at its active center. Based on this discovery, researchers, through meticulous design, successfully developed MOF-based nanozymes that can effectively simulate SOD activity. Hitomi et al. successfully developed a nanohybrid bimetallic zeolitic imidazolate framework, CuZn-ZIF-8 [60]. This material was composed of copper ions (Cu^2+^), zinc ions (Zn^2+^), and 2-methylimidazole ligands, where the Cu^2+^ and Zn^2+^ ions were bridged by the methylimidazole linkers. The CuZn-ZIF-8 exhibited SOD-like catalytic activity, primarily attributed to its porous structure and abundant copper active sites. Furthermore, the material demonstrated excellent cycling stability, showing promising potential for future applications. A two-dimensional Zn-ion-doped Mn-TCPP nanosheet was constructed by Shi et al., possessing dual superoxide dismutase- and catalase-mimicking activities (Figure 2C) [48]. The catalytic mechanism of this material operates as follows: First, the dismutation of superoxide anions occurs through single-electron transfer between Mn (III) and Mn (II) coupled with outer-sphere proton-coupled electron transfer. Subsequently, hydrogen peroxide is decomposed via dual-electron transfer between Mn (III) and Mn (V), involving an inner-sphere proton-coupled electron transfer pathway. Cellular assays demonstrated the unique anti-inflammatory and pro-biomineralization properties of the 2D MOFs, while animal studies further confirmed its potent anti-arthritic efficacy, suggesting a promising strategy for future multi-target anti-inflammatory treatment. The poor biocompatibility of MOF-based superoxide dismutase mimetics originates from their tendency to undergo rapid decomposition in simulated body fluids or lysosomal acidic environments, releasing organic ligands and metal ions [19]. This instability constitutes the primary cause of their inadequate biocompatibility and unpredictable toxicity profiles.

### 2.5. Hydrolysis Enzymes

Hydrolytic enzymes are a class of biomolecules with broad hydrolytic catalytic activity that efficiently and selectively cleave covalent bonds in substrates by activating water molecules as nucleophiles [61,62]. Research has shown that various MOF materials, such as Ce-based and Zr-based materials, can mimic the three main types of hydrolytic enzymes: esterases, proteases, and organophosphorus hydrolytic enzymes. The specific type of mimicry depends on the specificity of the substrate. Lou et al. reported a simple strategy and used 2-methylimidazole as the organic ligand to synthesize ultra-small Ce-MOFs with a size of approximately 5 nm at room temperature in aqueous solution (Figure 2D) [49]. This material exhibited excellent hydrolysis catalytic activity under mild conditions, effectively cracking phosphate bonds, glycosidic bonds and their mixtures, and degrading biofilms. The catalytic mechanism study showed that the synergistic effect between Ce^4+^/Ce^3+^ and the high-density active sites make the hydrolysis activity of this ultra-small Ce-MOFs 3 to 15 times higher than that of its bulk material. The catalytic activity of MOF-based hydrolase mimics is highly susceptible to strong acids and bases. This susceptibility primarily originates from the pH-dependent integrity of the MOF structure, the chemical state of the active sites, and the substrate-binding capacity. Strongly acidic or basic conditions directly compromise these critical functional elements [27].

### 2.6. Multi-Enzyme Assembly

Certain MOFs demonstrate two or more enzyme-mimetic activities under identical or varied reaction conditions. By integrating multiple catalytic functions within a single MOF structure, such materials can serve as efficient multi-enzyme mimics, offering an ideal platform for implementing complex cascade reactions and advancing biomedical applications [63,64]. Their catalytic mechanism relies either on the inherent multifunctional catalytic activity of the MOF itself, or on the integration of multiple natural enzymes or nanozymes onto the MOF scaffold to construct stable and efficient cascade systems. This strategy enables the highly sensitive and selective detection of target analytes [65]. Inspired by the compartmentalized catalytic organization in living cells, Li et al. proposed a biomimetic encapsulation strategy based on multi-shelled MOFs [66]. Using an epitaxial layer-by-layer overgrowth approach, they achieved spatially ordered organization and encapsulation of multiple enzymes within MOFs at the nanoscale. This design not only addresses the challenge of co-stabilizing incompatible enzymes, but also significantly enhances their synergistic catalytic efficiency. The experimental results demonstrated a 5.8–13.5-fold increase in the catalytic efficiency compared to free enzyme systems. Moreover, this method substantially reduces enzyme catalyst loading to the μg/mL range and has been successfully applied under mild conditions to co-encapsulate various cascade enzymes, including glucose oxidase, horseradish peroxidase, protease, and alcohol dehydrogenase. By constructing hierarchical enzymatic microdomains within a single MOF particle through a biomimetic approach, this work realized efficient synergistic catalysis and reaction integration among multiple enzymes, providing a new conceptual pathway for the rational design of highly efficient and tunable multi-enzyme cascade systems.

**Figure 3:**
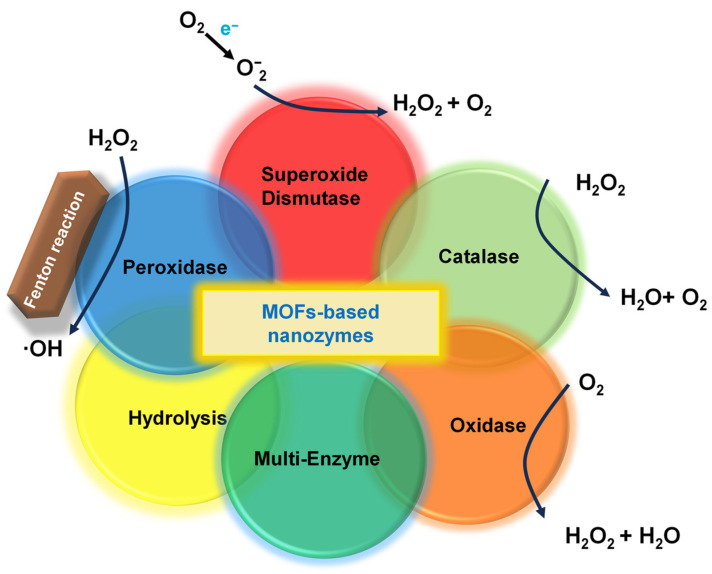
Schematic of the catalytic mechanism of representative MOF-based nanozymes.

As shown in Figure 3, the catalytic mechanism of representative MOF-based nanozymes is clearly revealed. The catalytic activity of MOF-based nanozymes primarily stems from two key mechanisms: first, transition metal ions, such as Fe, Cu, Co, Ni, and Ce, present in the structure can participate in redox reactions through changes in the oxidation state, thereby conferring enzyme-like catalytic activity to the material; second, specific organic ligands can mimic the catalytic process of natural enzymes, serving as electron transfer mediators to facilitate electron transfer between the substrates [18,67]. Compared to natural enzymes, these nanozymes typically offer advantages in terms of cost, stability, and overall catalytic activity. However, they exhibit lower substrate specificity, and their catalytic activity is significantly influenced by pH values (Table 2).

## 3. Synthesis of MOF-Based Nanozymes

**Figure 4:**
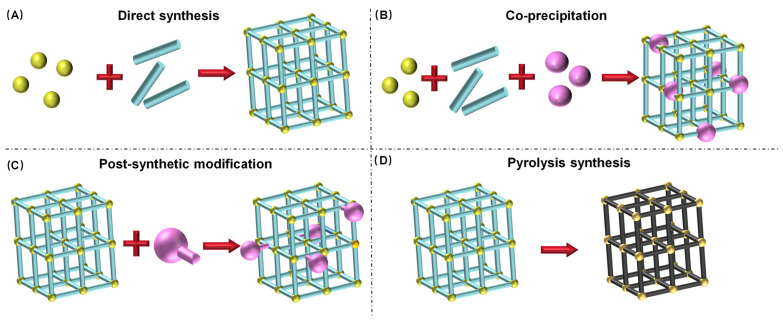
The primary synthetic strategies for MOF-based nanozymes, spanning (**A**) direct synthesis, (**B**) co-precipitation, (**C**) post-synthetic modification and (**D**) pyrolysis.

How to efficiently design nanozymes has remained one of the pressing challenges in the field. In recent years, extensive exploration of MOF-based nanomaterials for mimicking natural enzymes has provided novel approaches for constructing new nanozymes, particularly in the biomimetic design of active sites resembling those of natural enzymes. In this section, the design strategies for MOF-based nanozymes are summarized (Figure 4).

### 3.1. Direct Synthesis

MOFs represent a class of porous crystalline materials formed through the self-assembly of metal ions/clusters and organic ligands exhibiting distinct advantages, including tunable structures and tailorable functionalities [83,84]. Significantly, the transition metal nodes (e.g., Fe, Cu, Co, Ag, Zr, and Zn), which play pivotal roles in enzymatic metabolic processes, serve as the catalytic active sites [37,85]. When coordinated with organic ligands containing carboxyl, amino, phosphate, or sulfonate functional groups, these metal nodes form diverse active centers, resulting in highly ordered MOF materials with exceptional catalytic performance [86,87]. Synthetic methodologies for MOF-based nanozymes, including hydrothermal synthesis, microwave-assisted synthesis, and sonochemical synthesis, have been widely used. The following sections elaborate on each aspect respectively. The solvothermal method stands as one of the most classical and widely employed approaches for synthesizing MOF materials. By utilizing a sealed reaction system under elevated temperature and pressure, this technique facilitates the coordinated self-assembly of metal ions and organic linkers, yielding materials with high crystallinity, well-defined structures, and stable performance. For example, by using heme-mimetic TCPP ligands as the structural units coordinated with three transition metal nodes—Co, Cu, and Zn—as the organic linkers, a series of 2D M-TCPP nanosheets were constructed [88]. The resulting bimetallic two-dimensional Co-TCPP demonstrated superior catalytic activity toward H_2_O_2_ reduction.

Microwave-assisted synthesis converts electrical energy into kinetic energy through endogenetic heating mechanisms by exposing reaction mixtures to microwave radiation in the 300 GHz to 300 MHz frequency range. This enables the rapid and uniform heating of reaction systems, effectively accelerating nucleation. It has emerged as a valuable synthesis technique in the field of inorganic materials [89]. For instance, Meng et al. employed microwave-assisted synthesis to prepare MIL-101(Cu, Fe) nanozymes exhibiting excellent peroxidase-like activity, significantly reducing the reaction time to just 2 h [90]. Furthermore, microwave-assisted synthesis can optimize the performance characteristics of MOF materials, enhancing the technology’s significance for future development. In recent years, microwave-assisted synthesis has also been widely employed for the preparation of MOFs–fiber composites. Wang et al. synthesized ultrathin 2D Zr-BTB MOF nanosheets vertically on polypropylene fabric via a microwave-assisted solvothermal method [91]. The material consisted of six connected Zr_6_O_4_(OH)_4_^12+^ clusters and 1,3,5-tris(4-carboxyphenyl)benzene (BTB) linkers. Thanks to the more accessible active sites and lower diffusion barriers provided by the vertically grown and separated 2D MOF nanosheets, the composite fabric demonstrated not only excellent catalytic performance, but also a single-pass filtration detoxification efficiency of over 88%, along with good reusability. However, microwave synthesis also presents challenges related to safety and reproducibility.

Sonochemistry is a technology that utilizes ultrasonic cavitation effects to drive chemical reactions. During this process, bubbles are generated within a liquid, undergoing rapid formation, expansion, and collapse, inducing localized ultra-high temperatures (5000–25,000 K), high pressures, and extreme heating or cooling rates [92]. The impact of these extreme conditions on the acceleration of uniform nucleation and crystallization processes has been demonstrated to be significant, offering advantages such as reduced synthesis times, minimized energy consumption, and the facilitation of defect site formation. Ahn et al. synthesized Zr-based porphyrinic MOFs (MOFs-525 and MOFs-545) using a sonochemical method, achieving substantially reduced synthesis times of 2.5 h and 0.5 h, respectively [93]. In comparison, conventional solvothermal synthesis requires 18 h to produce MOFs-545 crystals. The sonochemically prepared MOFs-525 and MOFs-545 demonstrated improved catalytic performance in the hydrolysis of dimethyl-4-nitrophenyl phosphate, as well as a faster and greater adsorption capacity for bisphenol-A relative to samples obtained through traditional methods.

### 3.2. Co-Precipitation

The co-precipitation strategy, also known as the “one-pot synthesis” method, involves mixing natural enzyme solutions, pre-synthesized catalytic nanoparticles, and MOF species precursors, followed by their simultaneous co-precipitation within an MOF matrix, ultimately yielding MOF-based nanozyme composite materials [94]. This strategy has the advantage over step-by-step approaches of enabling the target product to be synthesized quickly and conveniently, effectively saving time and money. For instance, Yang’s group synthesized CeOx@fZIF by dispersing and restricting cerium oxide (CeOx) and fluorescence dye within the ZIF framework [95]. The obtained composites exhibited peroxidase-like activity. In the presence of alkaline phosphatase (ALP), the Cu (II) within the ZIF shell underwent reduction, precipitating structural collapse and subsequent unblocking of the shell. The synergistic and controllable recovery of both the chromogenic catalytic enzyme activity of the encapsulated CeOx and the dye fluorescence was enabled by this, thus constructing a dual-signal sensor capable of fluorescent and colorimetric detection.

**Figure 5:**
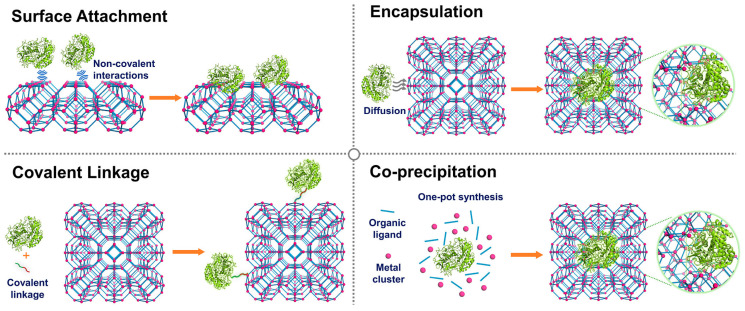
Strategies to prepare MOFs−enzyme composites, including surface attachment, encapsulation, covalent linkage, and co-precipitation [96].

Based on the core requirements for MOFs−enzyme composites (Figure 5), such as enzymatic activity, tolerance under harsh conditions, loading capacity, and recyclability, both specific and general construction strategies have been developed. Regarding the immobilization of enzymes with diverse cofactors involved in different reactions, four reliable main methods have been established [96]. For example, Ge’s group used a co-precipitation strategy to synthesize enzyme-amorphous MOFs (aMOFs) composites by packaging glucose oxidase (GO) in aMOFs at ambient conditions [71]. The aMOFs offer a satisfactory protective effect for the encapsulated enzyme, demonstrating enhanced stability in comparison with the native counterpart. The GO-aMOFs nanocomposites exhibit a 20-fold higher catalytic activity compared to their crystalline MOF counterparts. In addition, these materials enable noninvasive glucose monitoring in single living cells, thereby providing a novel technological platform for discriminating cancer cells from normal cells. This strategy is applicable for constructing nanozyme systems that require the incorporation of natural enzymes or other catalytic species, but are challenged by recycling constraints, aggregation tendencies, and poor dispersion.

Compared with other strategies, enzyme-encapsulated MOFs require more stringent, yet mild synthesis conditions, such as room temperature and aqueous solutions. The core of this approach lies in the direct encapsulation of enzymes into the preformed pores of MOFs. To achieve a high loading capacity and enhanced catalytic performance of the composites, precise matching between the MOFs’ pore apertures and the enzyme’s molecular dimensions is crucial [97]. By using cytochrome c, horseradish peroxidase, and lipase as templates and precisely regulating the structure of MOFs, Lv et al. successfully immobilized the enzymes within customized mesopores [98]. This approach not only avoided the inherent drawbacks of surface adsorption but also achieved selective size- and shape-complementary encapsulation of multiple enzymes. The catalytic activity of all the immobilized enzymes significantly exceeded that of their free and conventionally immobilized counterparts, with cytochrome c exhibiting the most prominent enhancement—an increase of over 1600%.

### 3.3. Post-Synthetic Modification

The post-synthetic modification of MOFs is a strategy that allows for the targeted functionalization of already synthesized MOFs using chemical methods, all while maintaining the integrity of the original framework [99,100]. This method utilizes reactions such as the formation of coordination bonds and the transformation of functional groups to enable the accurate integration of functional components and the controlled creation of intricate structures, ultimately embedding specific catalytic centers or functional groups within an MOF architecture [101,102]. Such a strategy provides a versatile and exact means of developing MOF-based nanozymes, enabling customized catalytic properties, selective activity, and improved stability, without undermining the structural framework [103].

Additionally, the efficiency and potential for scale-up inherent in this modification approach make it a favorable candidate for mass-producing MOF-based nanozymes. This strategy has been realized through several approaches, such as modifying the MOFs surface for catalytic substances via physical adsorption or covalent modification [104,105]. By using ZrFe-MOFs as the core, platinum metal precursors were in situ reduced to prepare ZrFe-MOFs@PtNPs (MOFs@Pt) [106]. In this system, the MOFs@Pt exhibited excellent peroxidase-like activity (SA = 21.77 U/mg), superior stability, and more modification sites. In comparison to physical adsorbing, covalent modification has been demonstrated to offer enhanced stability for the immobilization of catalytic species on MOFs [107]. By adsorbing glucose oxidase (GOx) on a boronic acid-functionalized hierarchically porous MIL-88B, HP-MIL-88B-BA was synthesized by Zhao’s group [108]. The HP-MIL-88B-BA has a layered porous structure. This structure not only provides abundant sites for the efficient fixation of Gox and prevents its leakage, but the hybrid nanocatalyst GOx@HP-MIL-88B-BA significantly improves the mass transfer efficiency of the matrix, thereby shortening the detection response time. However, post-synthetic modification still faces several critical challenges, including maintaining the structural integrity of MOFs, controlling the uniform distribution of functional groups, and reducing the yield resulting from multi-step reactions.

### 3.4. Pyrolysis Synthesis

MOF pyrolysis refers to a high-temperature treatment process where MOFs serve as the precursors under a controlled temperature and atmosphere [109]. This process induces the carbonization of organic linkers and triggers the reduction or transformation of metal nodes, ultimately yielding carbon-based composites or metal oxides. As a cornerstone strategy for MOF derivatization, pyrolysis significantly enhances the electrical conductivity, catalytic activity, and stability of the resulting materials, rendering it highly suitable for constructing high-performance nanozymes. Qu et al. reported a porous iron single-atom nanozyme (pFeSAN) with high oxidase-like activity [110]. Using iron-containing hemoglobin as both the template and iron source, they synthesized an MOF precursor in an aqueous medium. Subsequently, a straightforward one-step pyrolysis directly produced the mesoporous Fe-N_3_ single-atom nanozyme with an unsaturated coordination structure. The material’s high specific surface area and mesoporous architecture facilitate the full exposure of Fe-N_3_ active sites, substantially improving the site utilization and oxidase-mimicking activity. Its specific activity can reach 593 U mg^−1^, which is approximately 3.5 times and 8471 times higher than that of conventional Fe single-atom catalysts and Fe_3_O_4_, respectively. Leveraging this exceptional performance, pFeSAN enables the highly sensitive detection of millimolar-level glutathione (GSH) and allows for the visual analysis of GSH levels in tumor-associated cells and tissues.

## 4. Sensing Applications of MOF-Based Nanozymes

To date, a number of MOF-based nanozymes have been developed and applied in biosensing analysis. This section introduces the application of this kind of biosensors in bioanalysis.

### 4.1. Biomarker Detection

**Figure 6:**
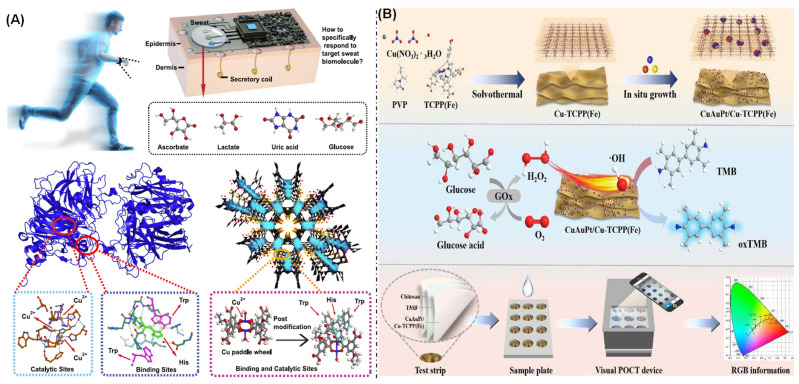
(**A**) Schematic of the biosensor for the detection of ascorbic acid [114]. (**B**) Schematic of the synthesis of CuAuPt-loaded Cu-TCPP(Fe) and glucose detection via colorimetric assay testing [116].

Biomarkers are defined as objectively quantifiable biological molecules, structures, or processes that serve as indicators of normal physiological states, pathogenic processes, or pharmacological responses to therapeutic interventions [111,112]. These quantifiable biological markers facilitate the identification of system dysfunctions, exposure to toxicants, or metabolic alterations, thereby providing critical tools for disease diagnosis, prognosis, and therapeutic monitoring in clinical medicine and toxicological assessment. Among the numerous biomolecules in sweat, ascorbic acid is one of the most important biomarkers associated with nutrition and immunity, playing an indispensable role in many bodily functions, such as iron absorption, collagen synthesis, infection protection, and the prevention of neurological disease [113]. Therefore, the detection of ascorbic acid in sweat is of great significance for assessing and preventing the risk of these diseases. Xia et al. prepared a copper-based MOF co-modified with tryptophan and histidine (HT-STAM-17-OEt) based on the natural structure of ascorbic acid oxidase using epitaxial growth and post-synthesis treatment methods (Figure 6A) [114]. This MOF single crystal exhibits the specific recognition and oxidation of ascorbic acid, capable of shielding interference from high concentrations of lactic acid, uric acid, and other sweat molecules. An HT-STAM-17-OEt-based ascorbic acid sensor operates via a mechanism in which amino acids induce the specific capture and recognition of ascorbate, followed by its rapid oxidation at copper active sites. It exhibits a linear dynamic range from 3 µM to 0.897 mM, a high sensitivity of 0.41 mA cm^−2^ mM^−1^, and a low detection limit of 1.0 µM (signal-to-noise ratio S/N = 3) in a neutral NaCl solution, enabling the accurate determination of ascorbate levels. Its performance far surpasses that of electrodes based on ascorbate oxidase. This work not only demonstrates an effective example of sweat ascorbic acid-specific detection but also provides important insights for exploring MOF-based nanozymes in the structural mimicry of natural oxidases. Chen et al. constructed a wearable sensor for detecting vitamin C and uric acid in sweat metabolites by fabricating a conductive MOF Ni_3_HHTP_2_ (HHTP = 2,3,6,7,10,11-hexahydroxytriphenylene) as a layered thin-film electrode on a flexible and breathable nanocellulose substrate [115]. Based on the intrinsic conductivity, highly porous structure, and excellent catalytic activity of the MOF-based layered film, the sensor exhibited outstanding performance on practical tests, with results that showed high consistency with those obtained by high-performance liquid chromatography. This study provides a feasible pathway for integrating multifunctional MOF materials into flexible electronic devices, thereby opening a new direction for the development and application of high-performance noninvasive biosensing technology.

Blood glucose is one of the most critical and universally measured biomarkers in the human body, playing an indispensable role in the screening, diagnosis, and daily management of diabetes. Recently, Ding et al. reported a CuAuPt/Cu-TCPP(Fe)-based colorimetric assay for the sensitive detection of glucose, achieving a detection limit of 4.0 μM. Furthermore, an integrated sensing platform comprising CuAuPt/Cu-TCPP(Fe)-based test strips and a smartphone was developed for point-of-care testing. This system holds promise for meeting the demands of personalized health monitoring and portable diagnostic devices (Figure 6B) [114]. Uric acid is the end product of purine catabolism and is usually regarded as a potential risk factor for various diseases [117]. Hyperuricemia characterized by elevated serum uric acid levels is closely related to various diseases, such as gout, cardiovascular diseases, and diabetes. At present, hyperuricemia and its related diseases are on the rise worldwide, especially in most developed countries. Zhang et al. reported that hemin/G4 DNA@Cu_2_O/Zr-MOF (HGCZ), which were co-incubated, exhibited excellent enzyme-like catalytic activity for the detection of uric acid in interstitial fluid [118]. Compared with Cu_2_O, Cu_2_O/Zr-MOFs had a larger specific surface area and enhanced enzyme activity. HGCZ, combining DNA and Cu_2_O/Zr-MOFs, had higher sensitivity and stability. This method could avoid natural uricase and reduce costs and has low requirements for environmental conditions during detection.

**Figure 7:**
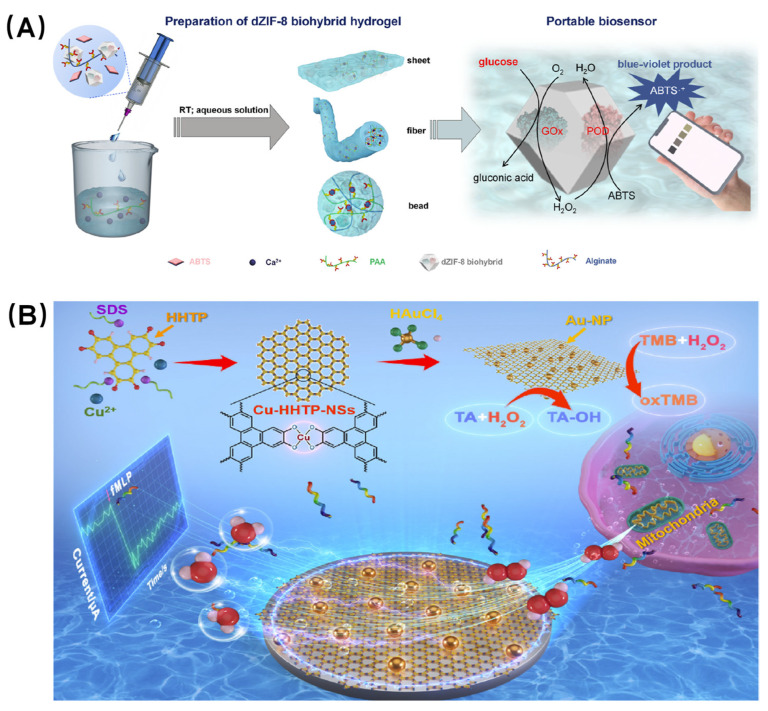
(**A**) Schematic of enzyme-encapsulated defective MOF hydrogel for glucose detection [119]. (**B**) Illustration of solvothermal synthesis of AuNPs/Cu-HHTP-NSs and its application in modified electrode for real-time tracking of H_2_O_2_ released from mitochondria in live human colon cells [120].

Figure 7A shows how Ouyang et al. co-encapsulated glucose oxidase and peroxidase within an MOF hydrogel. This system effectively converts glucose into a blue–violet product via the biocatalytic cascade reaction of the encapsulated enzymes. When integrated with a smartphone, it enables the sensitive and selective detection of glucose, exhibiting a linear range of 0.05 to 4 mM. Furthermore, the synergistic effect of the hydrophilic hydrogel and the MOF’s “armor” structure significantly enhances enzyme stability, allowing the sensor to maintain excellent sensing activity even after 30 days of storage at room temperature [119].

Hydrogen peroxide (H_2_O_2_) plays a crucial role in biological systems, and its level in the human body can serve as an important biomarker for the early diagnosis of various diseases, such as cancer. Electrochemical biosensors offer comprehensive advantages for H_2_O_2_ detection, including rapid response; high sensitivity; and the capability for miniaturized, online, and in vivo analysis [121]. As shown in Figure 7B, Sun et al. developed a high-performance electrochemical biosensor centered on a highly active dual-nanozyme signal amplification system [120]. This system utilizes ultrathin two-dimensional conductive MOF nanosheets (Cu-HHTP, where HHTP = 2,3,6,7,10,11-hexahydroxytriphenylene) as a support, which are loaded with high-density ultrafine gold nanoparticles (AuNPs). Benefiting from the synergistic effects of the dual-nanozyme activity of Cu-HHTP nanosheets and AuNPs, as well as their unique structural and electrical properties, the sensor exhibits outstanding detection performance for H_2_O_2_, with a low detection limit of 5.6 nM and a high sensitivity of 188.1 μA·cm^−2^·mM^−1^. Furthermore, the sensor was successfully applied to the real-time monitoring of H_2_O_2_ released from different human colon cells, enabling effective discrimination between colon cancer cells and normal colon epithelial cells. This achievement demonstrates the promising potential of and provides strong support for the application of the sensor for the early diagnosis and disease management of various cancers.

The integration of MOFs with polymer fibers has enabled the formation of fibrous composite materials that exhibit advantages over traditional single-component polymer films and mixed-matrix membranes [122]. MOFs–polymer fibrous composites not only facilitate more efficient molecular transport but also provide easier access to the active sites of MOFs. These characteristics render them promising for applications in detection and sensing fields. Similarly, for the in situ detection of H_2_O_2_, Huang et al. utilized a gradient porous hollow fiber membrane as a substrate, combined with MOF nanozymes and carbon nanotubes (CNTs), to construct a non-enzymatic electrochemical electrode with excellent performance [123]. This electrode demonstrated a rapid response, high selectivity, and strong stability. The introduction of CNTs not only provides a high specific surface area, promoting the uniform distribution of MOF-based nanozymes, but also establishes a three-dimensional conductive network. This network effectively facilitates electron transfer during the catalytic process, thereby overcoming the inherent poor conductivity of MOF materials.

Traditional biological markers and clinical symptoms and signs lack sufficient sensitivity and specificity to guide treatment decisions with respect to infectious diseases. Since the concentration of procalcitonin in the blood of patients with sepsis was first found to be significantly elevated in 1993, procalcitonin (PCT) has become an important biomarker for diagnosing bacterial infections [124]. More and more evidence has supported the idea that by detecting the concentration of serum procalcitonin, the types of infectious pathogens can be diagnosed early, the severity of infection can be evaluated, medication can be guided and the prognosis can be judged. Xie et al. prepared an oxidase-like nanozyme MnxOy@dPCN (MdP) by loading mixed-valence manganese oxide (Mn_x_O_y_) onto defective PCN-224 MOFs (dPCN) [125]. The mixed-valence state of manganese oxide gave the MdP redox and catalytic properties, and the high oxidase-like catalytic performance of MdP in the TMB substrate also resulted from its good electrical conductivity and affinity for the substrate. An immune biosensor was established for detecting the biomarker PCT based on the high catalytic properties of MdP nano-enzyme. A PCT immunoassay demonstrated satisfactory accuracy and repeatability, with a broad linear detection range of 0.05–100 ng mL^−1^ and a low detection limit (LOD) of 0.011 ng mL^−1^. These findings not only present a novel approach for developing oxidase-mimicking nanozymes but also establish a reliable method for sensitive PCT detection in complex matrices.

### 4.2. Bacterial Detection

Bacterial infections have always been a major challenge to global public health, and their early and precise detection is crucial for controlling their spread. However, traditional detection methods, such as microbial cultures, enzyme-linked immunosorbent assays, and polymerase chain reactions, have shortcomings, such as being time-consuming, having low sensitivity, and being dependent on expensive equipment, making it difficult to meet the demand for rapid on-site detection [126]. Therefore, developing sensitive, selective and simple rapid bacterial diagnostic methods to effectively prevent the worsening and spread of infections has become an important research direction. The emergence of nano-enzymes has provided a new alternative solution for the rapid diagnosis of bacterial infections.

Aiming at the problems that traditional bacterial detection methods take a long time and have difficulty achieving in situ sterilization, Bi et al. developed a new type of sensor. The sensor uses hollow ZIF-8-encapsulated glucose oxidase (GOx@hsZIF-8), whose porous structure boosts the enzyme activity by 5.2 times [127]. Meanwhile, specific recognition is achieved by combining aptamer-encoded silver nanoparticles (AgNPs). With the aid of catalytic hairpin assembly technology, the detection limit of this sensor is as low as 3 CFU/mL, and its linear range spans 10^7^ orders of magnitude. More importantly, the in situ generated Ag^+^ from the cascade reaction process can simultaneously achieve efficient sterilization, with a sterilization rate as high as 99.9% within 2 h, fully realizing detection and elimination. The sensor technology studied in this paper effectively resolves the contradiction between enzyme activity and stability, ingeniously combining the excellent mass transfer advantages of hollow MOFs with the high selectivity of aptamers, and providing a new idea for the design of multifunctional biosensors.

In a recent study, Yang et al. developed a novel nanozyme mimetic, MoO_3_/MIL-125-NH_2_, and coupled it with a bacteriophage to serve as a probe for the highly sensitive and specific detection of staphylococcus aureus [128]. The construction of a nanozyme material with significant peroxidase-like activity was achieved by combining MoO_3_ with MIL-125-NH_2_. Research has demonstrated that the synergistic interaction between these two components effectively promotes the generation of superoxide radicals (O_2_•^−^), thereby significantly enhancing the efficiency of the H_2_O_2_-mediated TMB oxidation reaction. The proposed biosensor with optimized detection capabilities is capable of achieving a very low limit of detection of 16 CFU mL^−1^, with a wide linear range from 10^1^ to 10^8^ CFU mL^−1^. In conclusion, this phage-based MOF enzyme biosensor demonstrates several advantageous properties, including ultrasensitivity, high selectivity, and good reproducibility. This work provides a foundation for further research into phage-based detection methods.

Compared with single-atom MOF-based nanozymes, diatomic nanozymes exhibit an astonishing synergistic effect on catalytic activity. Hosseini et al. developed a groundbreaking bimetallic Zr-Pr MOFs (ZPR-MOFs) nanozyme platform for the highly sensitive detection of *Salmonella typhimurium* (*S. typhimurium*) [129]. The ZPR-MOFs exhibited remarkable peroxidase-like activity, efficiently catalyzing the oxidation of TMB by H_2_O_2_ to generate a distinct colorimetric response. In conditions that are optimal, with regard to pH and temperature, the biosensor is capable of detecting *S. typhimurium* in the range of 10^2^–10^8^ CFU/mL, with an LOD of 37 CFU/mL.

**Figure 8:**
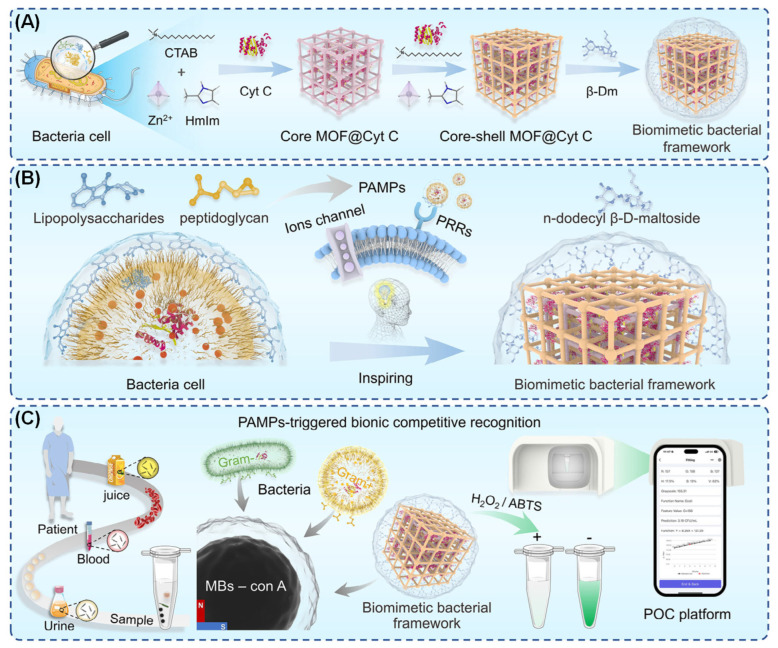
Schematic of the synthetic process of biomimetic bacterial framework and point-of-care testing for bacterial detection [130]. (**A**) Schematic illustration of the synthetic route of MOF@Cyt C, MOF@Cyt C and BBF. (**B**) Schematic illustration of the design inspiration and rapid recognition mechanism of BBF. (**C**) The working principle of the POCT platform from sample to result. “+” indicates the presence of bacteria, whereas “−” indicates their absence.

The ability to swiftly and accurately detect bacteria is of paramount importance for the preservation of public health. In this regard, Yang et al. developed a biomimetic bacterial framework (BBF, Figure 8A) for the instantaneous, broad-spectrum detection of bacteria, inspired by bacterial cell structures [130]. The BBF was constructed as follows: First, cytochrome c oxidase (Cyt C) was encapsulated within a CTAB-regulated MOF to form the core MOFs@Cyt C. This was then subjected to heterogeneous accelerated nucleation for secondary encapsulation, constructing the core–shell structure MOFs@Cyt C. Finally, the precursor underwent glycosylation modification using dodecyl-β-D-maltoside (β-Dm) to yield the BBF. This bioinspired framework exhibits dual biomimetic functions: it mimics the synergistic catalytic signal amplification capability of bacterial extracellular and intracellular enzymes, whilst its β-Dm polysaccharide shell mimics bacterial pathogen-associated molecular patterns, thereby enabling rapid recognition by pattern recognition receptors (Figure 8B). During the process of detection, magnetic bead-modified pattern recognition receptor proteins are capable of simultaneously recognizing both native bacteria and the BBF. In the presence of native bacteria in a sample, a competitive interaction ensues between the bacteria and BBF for receptor protein binding, thereby impeding the BBF’s signal catalysis. This inhibition induces a distinct color change in the catalytic substrate, thereby generating the detection signal output. The system is integrated with a smartphone running a customized application and 3D-printed equipment. This integration enables the rapid, sensitive detection of Gram-negative and Gram-positive bacteria within 40 min. The detection limits for these samples were found to be 10^2^ CFU mL^−1^ and 10^3^ CFU mL^−1^, respectively, thereby meeting the requirements for point-of-care testing, as outlined in Figure 8C. Table 3 shows some of the reported MOF-based nanozymes biosensors for the detection of different pathogens.

### 4.3. Virus Detection

**Figure 9:**
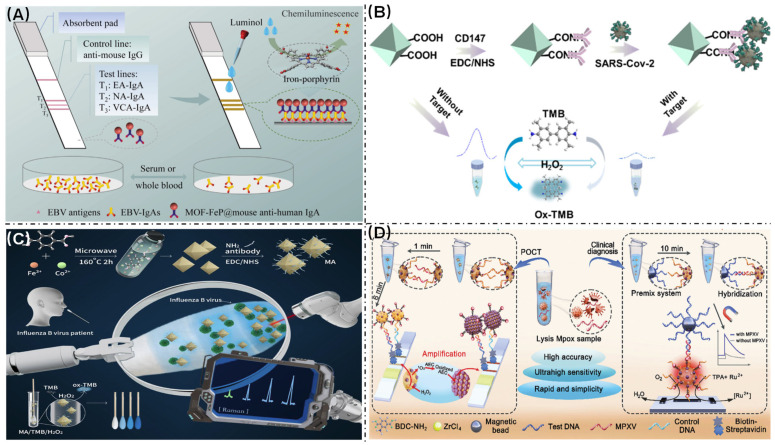
(**A**) Principle of as-developed MOFs-FeP single-atom nanozymes for EBV antibodies rapid detection [141]. (**B**) Schematic illustrating preparation process of SARS-CoV-2 immunosensor based on MIL-101(CuFe) [90]. (**C**) Principle of detecting influenza virus from nasopharyngeal swab samples using MIL-101(CoFe) [142]. (**D**) Colorimetric–electrochemiluminescence dual-mode biosensor for detecting SARS-CoV-2 S-protein [143].

At present, the spread of various viruses has had negative effects on human life and health. It has become crucial to develop enhanced virus diagnostic technologies to mitigate future outbreaks. The correlation between Epstein–Barr virus (EBV) infection and an increased risk of nasopharyngeal carcinoma (NPC) is well established [140]. To improve the efficiency of EBV screening at an early stage, researchers such as Yan et al. have developed a new detection technology based on single-atom nanozymes (Figure 9A) [141]. The team successfully synthesized single-atom nanozymes that can precisely mimic the catalytic sites of natural peroxidase using an iron porphyrin-based metal–organic framework (MOF-FeP) and integrated them into traditional test strips to create a highly sensitive EBV-IgA rapid detection platform. Compared with the current ELISA, the MOF-FeP test strip offers several advantages: it provides a greater detection sensitivity, ranging from 75.56% to 93.30% compared with 64.44% to 82.22% for ELISA, and it reduces the detection time from 1 to 2 h to just 16 min. Combining the ease of operation of traditional test strips with the high catalytic activity of single-atom nanozymes, this new detection tool provides an efficient, immediate EBV-related disease screening strategy with significant potential for clinical transformation.

COVID-19 is a highly contagious disease caused by the novel SARS-CoV-2 virus. During its continuous global spread over the past three years, the virus has rapidly mutated into multiple branches, posing higher requirements for detection technology. This technology must be accurate, rapid, and low cost in order to enable early screening and timely intervention. In response to this demand, Meng et al. developed an efficient detection method based on nanozymes (Figure 9B) [90]. They introduced Cu atoms into MIL-101(Fe) to create MIL-101(CuFe), and discovered that the combined effect of Cu and Fe could greatly enhance the material’s peroxidase-like activity, catalyzing the decomposition of H_2_O_2_ to generate hydroxyl radicals (•OH). These radicals then oxidized the chromogenic reagent TMB to produce a chromogenic signal. The research team achieved the efficient capture of the virus by modifying the specific CD147 protein on the nano-enzyme surface and exploiting its specific binding to the spike protein on the novel coronavirus surface. When the virus was bound to the nano-enzyme surface, its catalytic activity was inhibited, resulting in a weaker color reaction. This principle forms the basis of a detection method that could complete qualitative and quantitative analyses of SARS-CoV-2 within 10 min, with a detection limit as low as 3 PFU/mL, demonstrating outstanding sensitivity.

Meng et al. developed a bimetallic MIL-101(CoFe)-based colorimetric and surface-enhanced Raman scattering (SERS) dual-mode biosensor for the rapid and ultrasensitive detection of influenza virus (IBV) (Figure 9C) [142]. Leveraging the synergistic catalytic effect of Co and Fe dual-metal centers, the nanozyme exhibited significantly enhanced peroxidase (POD)-like activity, efficiently catalyzing the substrate TMB by H_2_O_2_ to generate blue oxidized TMB (ox-TMB) and its characteristic Raman fingerprint, thereby achieving dual-signal amplification. Upon functionalization with influenza virus-specific monoclonal antibodies (Anti-FluB-MmAb), the biosensor demonstrated the selective capture of influenza virus from nasopharyngeal swab samples, enabling visual screening within 5 min and ultrasensitive quantitative detection via SERS, with a remarkable LOD of 1.3 ng/mL within 5 min.

Yang et al. developed an innovative bifunctional nanoprobe, Ru@U6-Ru/Pt NPs, utilizing a zirconium-based metal–organic framework (UiO-66-NH_2_) as a carrier platform for the colorimetric–electrochemiluminescence (ECL) dual-mode detection of monkeypox virus nucleic acids (Figure 9D) [143]. This sophisticated detection system integrates both ECL luminophores and peroxidase-like nanozyme PtNPs within an MOF matrix, achieving synergistic signal amplification while maintaining excellent stability. The visual detection LOD is 0.1 pM, with the ECL quantification mode achieving ultrasensitive detection with an impressive LOD of 10 aM. In clinical testing (*n* = 50), the platform exhibited complete concordance with standard qPCR assays, while achieving a significantly reduced turnaround time of 15 min.

### 4.4. Mycotoxins and Antibiotics Detection

**Figure 10:**
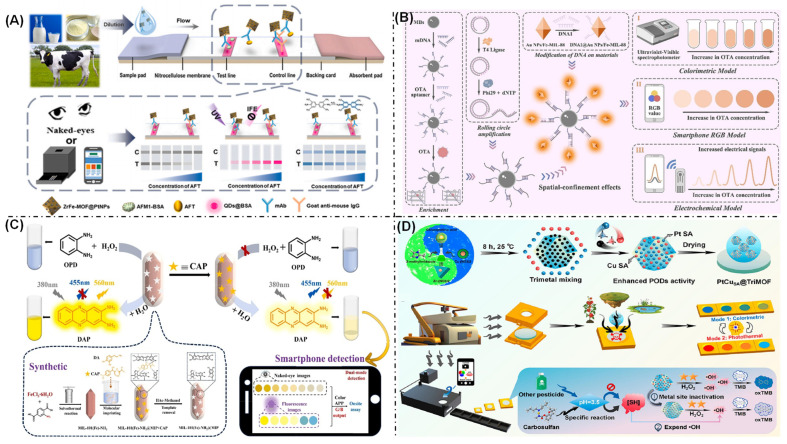
(**A**) Schematic of development of ZrFe-MOFs@PtNPs-based lateral flow immunoassay for multi-modal detection of aflatoxins via colorimetric, fluorescent, and catalytic readouts [106]. (**B**) Schematic of aptasensor for OTA based on Fe-MIL-88 nanozymes by rolling circle amplification for effective signal enhancement [144]. (**C**) Principle of ratiometric fluorescent–colorimetric dual-mode sensing approach for CAP detection based on MIL-101(Fe)-NH_2_@MIP nanozyme [145]. (**D**) Schematic of PtCuSA@TriMOFs nano-enzymes for multi-mode detection of carbosulfan with high detection specificity [146].

Mycotoxins are toxic secondary metabolites produced by filamentous fungi during crop cultivation, post-harvest storage and food processing. Due to their toxicity, carcinogenicity, and resistance to degradation, mycotoxins have prompted the development of numerous MOF-based detection assays and applications. Jiang et al. developed a sensitive and portable lateral flow immunoassay based on a ZrFe-MOFs@PtNPs nanocomposite for the detection of carcinogenic aflatoxins (Figure 10A) [106]. This method enables colorimetric, fluorescent, and catalytic multi-signal readouts, significantly improving the detection sensitivity to as low as 0.0062 ng/mL, which is two orders of magnitude lower than that of AuNPs-LFIA (0.1839 ng/mL). The prepared biosensor can detect AFM1 in milk and milk powder samples, with a recovery rate ranging from 102.71% to 115.25%. These results confirm that this method could be more widely applied in point-of-care biosensors. Ochratoxin A (OTA), a prevalent mycotoxin, is widely found in various foodstuffs, such as grains, dried fruits, and fruits, and poses a significant threat to human health. Hu et al. developed an aptasensor for OTA based on Fe-MIL-88 nanozymes, which enables the quantitative detection of OTA through measurements of electrical current response, color variation, and absorbance changes (Figure 10B) [144]. Innovatively, this sensor utilizes the spatially confined effect generated by rolling circle amplification to effectively increase the local concentration of Fe-MIL-88 signal probes, thereby significantly enhancing its detection sensitivity. The method is operationally straightforward and exhibits both high sensitivity and selectivity, allowing for OTA detection across a wide concentration range, from 1 fg/mL to 250 ng/mL, with a detection limit as low as 0.22 fg/mL. In practical sample analyses, the results obtained from corn, wheat, and red wine samples demonstrate strong consistency with those from a commercial ELISA kit.

Chloramphenicol (CAP) and its analogs are highly effective broad-spectrum antibiotics extensively used in aquaculture and livestock farming. However, due to their high toxicity, particularly the hazards posed to the liver and hematopoietic system, many countries have imposed restrictions on their use. Li et al. successfully synthesized an MIL-101(Fe)-NH_2_@MIP composite material by in situ polymerization on the surface of MIL-101(Fe)-NH_2_ using molecular imprinting technology (Figure 10C) [145]. This material exhibits peroxidase-like activity and specific recognition capability toward CAP. The dual-functional MIL-101(Fe)-NH_2_@MIP probe prepared with this strategy provides a ratiometric fluorescent–colorimetric dual-mode sensing approach for CAP detection, achieving limits of detection (LOD) as low as 36.45 nM (fluorescent mode) and 93.38 nM (colorimetric mode), respectively. Successful spiked recovery tests in fresh milk samples have further validated the potential practical application value of this method. This research not only expands the application scenarios of MOF-based nanozymes, but also paves the way for the rapid and sensitive detection of antibiotics.

### 4.5. Pesticide Residues Detection

The quality and safety of agricultural products directly impact the safety of the food on peoples’ tables. Pesticide residues stand as one of the major factors affecting agricultural product safety. As a global public environmental issue, pesticide residues have become a primary challenge in the field of food safety. Therefore, developing specific in situ detection technologies for pesticide residues is of significant importance for ensuring food safety and enabling scientific pesticide management. Ye et al. developed a portable, intelligent, flexible sensor based on a trimetallic organic framework-derived Pt-Cu dual-atom nanozyme (PtCuSA@TriMOFs) (Figure 10D) [146]. The PtCuSA@TriMOFs effectively reduced the energy barrier for co-catalytic reduction in H_2_O_2_, thereby catalyzing the oxidation of TMB to generate dual-mode colorimetric and photothermal signals. The specific detection of the pesticide carbosulfan is achieved via its acidic hydrolysis. Upon hydrolysis, carbosulfan generates sulfide ions (SH^−^), which scavenge the hydroxyl radicals (•OH) in the system. This scavenging inhibits the catalytic activity of the Pt-Cu dual-atom active sites. This dual-inhibition mechanism (scavenging •OH and inhibiting catalytic activity) ensures high detection specificity. The sensor achieved detection limits of 4.2 nM for the colorimetric mode and 5.7 nM for the photothermal mode. This work provides a solid theoretical foundation for the in situ specific detection of pesticide residues resulting from spraying, thereby addressing food safety concerns. K. Farha et al. employed isothermal titration calorimetry, leveraging its capacity to detect minute differences in thermodynamic parameters to systematically obtain and compare the complete thermodynamic profiles of the interactions between various MOF materials and the model organophosphorus molecule ethyl phosphonic acid [147]. This study laid an important theoretical foundation for the development of MOF materials with superior catalytic performance.

## 5. Summary and Prospects

This article reviews the research progress in the field of MOF-based nanozymes in recent years, covering developments such as the expanding number of enzymes types, the deepening of our understanding of various reaction mechanisms, and the regulation of catalytic performance. MOF-based nano-enzymes are increasingly being widely used in fields such as biosensing due to their simple preparation methods, good stability, and easy separation and purification.

Although significant progress has been made in the research on MOF-based nanozymes, this field still faces many key challenges and technical bottlenecks that urgently need to be broken through:

(1) Although the catalytic activity of some MOF-based nanozymes is similar to, or even higher than that of natural enzymes, the catalytic activity of most nano-enzymes is still much lower than that of the corresponding natural enzymes. In addition, MOF-based nanozymes also have the problem of poor substrate selectivity. A great deal of effort will be needed in the future to rationally construct new types of nano-enzymes with high matrix selectivity and catalytic efficiency.

(2) Thus, the research focus on MOF-based nanozymes has mainly been on oxidoreductase, peroxidase, and catalase; relatively few studies have been conducted on other enzymes. Developing more types of nano-enzymes and exploring their cascade reaction units will deepen our understanding of natural enzymes and complex life processes. For instance, we could create analogs of natural enzyme active centers and then introduce them into MOFs or other nanomaterials to simulate catalytic activity.

(3) At present, the catalytic mechanisms of many nano-enzymes remain unclear. Further exploration of catalytic kinetics and mechanisms may help regulate the catalytic activity of nano-enzymes.

(4) MOF-based nanozymes are furthering the development of biosensing in the direction of high sensitivity and intelligence, mainly focusing on laboratory research. Their industrial applications still require breakthroughs in standardized preparation and toxicity evaluation.

(5) The development of data-inspired material design strategies, combined with machine learning, is expected to guide the synthesis and rational modification of enzyme-active materials, promoting the rapid development of the MOF-based nanozymes field.

(6) The synthesis of many high-performance MOF-based nanozymes often relies on harsh processing conditions. This directly results in significant batch-to-batch variations, making it challenging to maintain uniformity in both the structure and performance of the materials. A key direction for future development lies in devising novel synthetic strategies that are more environmentally benign, employ milder conditions, and are readily scalable.

(7) The catalytic durability of MOF-based nanozymes is often compromised by the instability of their frameworks in complex aqueous environments (e.g., extreme pH, competitive ligands), where bond hydrolysis or exchange causes collapse and activity loss. Advancing their practical use requires strategies to reinforce active-site anchoring and incorporate self-regenerative functions.

## Figures and Tables

**Table 2:** The main classification of MOF-based nanozymes.

Classification	Advantages and Limitations	Representative Materials	Reaction	Ref.
**Peroxidase**	Higher catalytic activity, but only in weak acidity conditions (pH about 4).	MOFs 808, Fe-PCN-222, Ni-MOFs	Fenton-like reaction	[68,69,70]
**Oxidase**	Higher catalytic activity, but the selectivity and specificity of substrate are insufficient in complex samples.	Ce-MOFs, D-ZIF-67, Cu-MOFs	ROS	[71,72,73,74,75]
**Catalase**	High stability, but only at specific pH.	Ce-MOFs, Mn-MOFs	Disproportionate decomposition	[76,77]
**Superoxide dismutase**	Higher stability, high catalytic activity, but poor biocompatibility.	Cu/Zr-MOF 818, Sn-PCN222	Superoxide anion disproportionation	[78,79]
**Hydrolase**	Higher stability, but activity of catalyst is affected by strong acids and bases.	MOFs-808, Ce-MOFs	Hydrolysis of metal nodes and coordination structures	[80,81,82]

**Table 3:** MOF-based nanozymes for bacterial detection.

Detected Bacteria	MOF Type	MOF Synthesis	Principle	Linearity Range	LOD	Ref.
*Staphylococcus aureus*	MoO_3_/MIL-125-NH2	Solvothermal	Bacteriophages-specific recognition	10^1^–10^8^ CFU/mL	16 CFU/mL	[128]
*E. coli* O157:H7	AgPt/PCN-223-Fe	Solvothermal	Antibody-modified MOFs	10^3^ to 10^8^ CFU/mL	276 CFU/mL	[129]
*S. typhimurium*	ZrPr-UIO-66	Solvothermal	Aptamer-modified MOFs	10^2^–10^8^ CFU/mL	37 CFU/mL	[131]
*Staphylococcus aureus*	Cu-MOFs	Solvothermal	Aptamer-modified Cu-MOFs	50 to 10 000 CFU/mL	20 CFU/mL	[132]
*Vibrio parahaemolyticus*	Fe_3_O_4_@MOFs(Fe-Cu)-GNS-MBA	Solvothermal	Aptamer-modified Cu-MOFs	10^1^–10^5^ CFU/mL	9 CFU/mL	[133]
*E. coli* and *Staphylococcus aureus*	AuAg@PB MOFs	Stirring at room temperature	4-mercaptophenylboronic acid conjugated bacteria	10^1^–10^9^ CFU/mL 10^1^–10^8^ CFU/mL	2 CFU/mL	[134]
*E. coli* O157:H7	AuPt/PCN-224	Solvothermal	Aptamer-modified MOFs	10^1^–10^6^ CFU/mL	10 CFU/mL	[135]
*S. typhimurium*	AuNPs@CuZr-MOFs	Solvothermal and stirring	DNA probe	3.5 to 3.5 × 10^6^ CFU/mL	0.82 CFU/mL	[136]
*Vibrio parahaemolyticus*	UiO-66	Solvothermal	Aptamer-modified MOFs	10^1^–10^7^ CFU/mL	4 CFU/mL	[137]
*Pseudomonas aeruginosa*	Cu-ZrMOFs	Solvothermal	Aptamer-modified MOFs	10^1^–10^6^ CFU/mL	2 CFU/mL	[138]
*Vibrio parahaemolyticus*	Fe_3_O_4_@ZIF-8 and Pt@ZIF-8	Solvothermal	Aptamer-modified MOFs	10^2^–10^7^ CFU/mL	15 CFU/mL	[139]

## Data Availability

This review article does not incorporate any primary research findings, software, code, or original data. No new data were generated or analyzed for this study.
